# Nurses' Experiences of Nonpatient Factors That Affect Nursing Workload: A Study of the PAONCIL Instrument's Nonpatient Factors

**DOI:** 10.1155/2014/167674

**Published:** 2014-06-18

**Authors:** Lisbeth Fagerström, Paula Vainikainen

**Affiliations:** ^1^Nursing Science, Faculty of Health Sciences, Buskerud and Vestfold University College, Papirbredden, Grønland 58, 3045 Drammen, Norway; ^2^Åbo Akademi University, Vaasa, Finland; ^3^Department of Musculoskeletal Diseases, Turku University Hospital, Turku, Finland

## Abstract

In the RAFAELA patient classification system, the professional assessment of optimal nursing care intensity level (PAONCIL) instrument is used to assess the optimal nursing intensity level per unit. The PAONCIL instrument contains an overall assessment of the actual nursing intensity level and an additional list of central nonpatient factors that may increase or decrease the total nursing workload (NWL). The aim of this cross-sectional study was to assess and determine which nonpatient factors affect nurses' experiences of their total NWL in both outpatient settings and hospitals, as captured through the PAONCIL instrument. The data material consisted of PAONCIL questionnaires from 38 units and 37 outpatient clinics at 11 strategically selected hospitals in Finland, and included nurses' answers (*n* = 1307) to the question of which factors, other than nursing intensity, affect total NWL. The methods for data analyses were qualitative content analyses. The nonpatient factors that affected nurses' experiences of total NWL are “organization of work,” “working conditions,” “self-control,” and “cooperation.” The actual list of nonpatient factors in the PAONCIL instrument is to a reasonable extent relevant, but the list should be improved to include nurses' actual working conditions and self-control.

## 1. Introduction

Nurse staffing is an actual problem and challenge in many countries, and nursing workload (NWL), nursing intensity (NI), and unattended patients' needs are organizational factors that significantly affect the quality of care and patient outcomes [1–3]. One prerequisite for the sustainable development of a health care system is the optimal allocation of care resources, in both quantity (number of nurses) and quality (competence; [4]). Thus, it is of crucial importance for health care outcomes that nurse managers be able to precisely staff units and systematically steer total NWL.

Patient classification systems (PCS), also known as patient acuity or nursing demands systems, have been developed to manage workloads and estimate the need for nursing resources through the identification and quantification of patient care needs [5, 6]. When discussing systems that are used to measure NWL and NI, the terms PCS and nursing demand methods are used. PCS is a generic term that encompasses any grouping of patients according to diagnosis, treatment, diagnosis related group, blood group, demographic factors, and so forth [7]. The term can also refer to systems used for documentation, the classification of interventions, or the categorization of patients according to an assessment of their nursing care requirements over a specified period of time [8]. According to Arthur and James [5], nursing demand methods is a term that includes any system used to determine the number and/or mix of nursing staff. While nursing demand methods commonly include an instrument to measure workload, they do not typically include the measurement of nonpatient-related (nonpatient) factors.

Measuring NI and NWL is a complex process, and many nonpatient factors affect nurses' NI and their experiences of total NWL. NI measures direct and indirect patient-related workload and does not include nurses' unit-related workload [9]. Measuring NI involves an assessment of the nursing activities and required level of nursing competence that patients need [7, 10–12]. NI can be described in terms of four dimensions: how sick a patient is, the patient's dependency on nursing, the nursing process, and the time required for nursing activities [7].

No common definition of NWL is seen in the literature. NWL measurement refers to any attempt to assess the total volume and/or level of nursing work [5]. According to a review study by Myny et al. [13], the main influencing variables related to NWL are the patient/family, nursing team, individual nurse, unit and hospital, and meta-characteristics, in other words, patient-related nursing activities, unit-related activities, and other organizational and individual-related factors. Still, a reliable picture of total NWL necessitates an assessment of central nonpatient factors that may affect nurses' experiences of their total NWL, for example, occupational stress, emotional exhaustion, intrinsic work motivation, and job satisfaction, and the personal characteristics of the individual [14, 15]. Factors related to the surrounding context and organization also have a clear effect on nurses' experiences of total NWL, such as physical and mental stress factors in the work environment, work tasks, the organization of work, and leadership in relation to personnel administration and/or culture [16].

Therefore, on the basis of the aforereferenced literature and for the purposes of this study, total NWL is defined as the sum of direct and indirect patient-related nursing activities, nonpatient nursing activities, and central nonpatient factors: those personal/nurse-related, contextual, and/or organizational factors may affect nurses' experiences of their NWL.

The RAFAELA PCS was developed in Finland in the late 1990s and is used to assess the need for nursing resources. RAFAELA was tested in 14 Finnish hospitals during 2000–2002 [17]. According to Carr-Hill and Jenkins-Clarke [18], approaches to measuring NWL can be classified into four groups: dependency-driven, task-oriented, care plan-driven, and unit-based. RAFAELA is a dependency-driven method with a bottom-up management approach (cf. [5]). The main goal of RAFAELA is to balance patients' care needs and nursing resources [19, 20]. Almost every Finnish hospital has now implemented this system and implementation is ongoing in Norway, Iceland, Sweden, and Holland. RAFAELA is composed of two instruments. The first is the Oulu patient classification (OPCq) instrument, whereby each patient's NI is measured daily. The second is the professional assessment of optimal nursing care intensity level (PAONCIL) instrument, which links NI to the use of resources and also provides information on some central nonpatient factors (12 in total) that may influence nurses' experiences of NWL.

In order to improve RAFAELA, and given the continuously changing health care environment, an assessment of the PAONCIL instrument in regard to its list of nonpatient factors was deemed necessary. The aim of the study was to assess and determine which nonpatient factors, seen under the factor “other factors” in the PAONCIL instrument, affect nurses' experiences of their total NWL in both outpatient settings and hospitals.

## 2. The OPCq and PAONCIL Instruments in RAFAELA

In RAFAELA, the OPCq instrument is used to measure daily NI, which is measured through the following six subareas: (1) planning and coordination of nursing care; (2) breathing, blood circulation, and symptoms of disease; (3) nutrition and medication; (4) personal hygiene and secretion; (5) activity, sleep, and rest; (6) teaching, guidance in care and follow-up care, and emotional support [10]. The NI can vary for each subarea from 1 to 4 points. The points are added up, giving a range of 6–24 NI points per patient. The total sum of NI points for all patients in the unit is then calculated, for example, 240 points. Then, the total sum of NI points for a unit is divided by the total number of nurses who had nursed the patients in the unit during that calendar day (e.g., 10 nurses). As seen in our example, the actual NI level would be 24 NI points per nurse.

The PAONCIL instrument is composed of two parts; the first part links NI to the optimal use of resources whereas the second part provides information on some central nonpatient factors. In the first part of the instrument, nurses assess the actual NI of a unit's patients [21] using a scale from −3 to +3 over a 4–6-week period. Every nurse makes an overall assessment at the end of each shift or before leaving the department, which determines whether the nursing resources have been sufficient in relation to patients' needs and NI. A PAONCIL score of zero is considered optimal and indicates that NI is balanced with patients' needs and that nurses have a realistic opportunity to provide good quality care [15, 22]. The optimal staffing level of a unit can be established through the linear regression analysis of the daily mean of the OPCq points per nurse and the daily mean of nurses' PAONCIL scores for the same period [21–23].

The second part of the instrument includes questions that reveal nonpatient factors that may increase or decrease nurses' experiences of NWL during a specific work shift. While the first version of the PAONCIL instrument, developed as a pilot-study in 1996, did not include nonpatient factors [23], nonpatient factors were included in the PAONCIL instrument and tested by methodological triangulation in 1999-2000 at two hospitals (11 factors; [24]). After this validity study in 1999-2000, the list of nonpatient factors was completed with the factor “Meetings and training.” Since 2002, the PAONCIL instrument includes the following 12 factors related to total NWL during a shift: (1) organization of work; (2) planning of work schedules; (3) staff substitutes; (4) meetings and training events; (5) nursing students; (6) cooperation with physicians; (7) cooperation with other staff; (8) cooperation within the organization, for example, different units; (9) cooperation with own group; (10) own work capacity (e.g., tiredness flu, and worries); (11) mental stress (e.g., terminal treatment and resuscitation); (12) other factors. Answer options include “increased workload” or “decreased workload”; no answer is interpreted as “no effect.” In 2002, the impact of the PAONCIL instrument's 12 nonpatient factors on NWL was statistically analyzed in 22 somatic units [15]. The study showed that OPC (=NI), mental stress, cooperation/coordination with other staff groups, meetings/training, cooperation with physicians, and other factors (seen in the PAONCIL list under “other factors”) were clearly independent explanatory variables for the nurses' total NWL. After this study, the decision was made to analyze in greater detail the content of the factor “other factors” to see whether the actual PAONCIL list of nonpatient factors should be revised and/or supplemented with new nonpatient factors.

## 3. The Aim of the Study

The aim of this study was to assess and determine which additional factors (again, reported under “other factors” in the PAONCIL list) affect nurses' experiences of their total NWL in both outpatient settings and hospitals. The overall purpose of the study was to therefore reassess the PAONCIL list of nonpatient factors and to assess, emanating from the study results, whether new nonpatient factors should be added to this list.

Ethical guidelines for research in health sciences [25] guided the conduct of this study. Each hospital gave permission to use the data collected for scientific purposes, and the data material used in this study was collected in connection with the PAONCIL assessment of optimal NI during the implementation or ordinary use of RAFAELA.

## 4. Methods

### 4.1. Sample

The organizations in this cross-sectional study were selected from the RAFAELA national database, which is maintained by the Finnish Consulting Group Ltd. [17]. We decided to select the sample from those units that conducted PAONCIL studies during 2010-2011. At that time, a total of 22 hospitals used RAFAELA and had conducted a PAONCIL study and recorded their results electronically. The purpose was to include hospitals of different sizes and to collect a large amount of data. Our assumption was that the size of an organization might affect nurses' experiences of the factors that affect their NWL. The hospitals were stratified into three groups: small scale (local hospitals), middle scale (central hospitals), and large scale (large central hospitals and university hospitals). Due to the limited number of small scale hospitals (*n* = 2), all were included in the study. A random sampling procedure was applied with regard to the middle and large scale hospitals; that is, every other middle scale hospital (*n* = 6) and every third large scale hospital (*n* = 3) were selected from a list and included in the study, resulting in a total of 11 hospitals. From these, 37 units and 38 outpatient clinics were included and 1 275 nurses (914 from units and 361 from outpatient clinics) participated in the study.

### 4.2. Material and Data Collection

All PAONCIL questionnaires (*N* = 22176) gathered during January 2010-September 2011 on the aforementioned units included in this study and which contained comments on question number twelve of the nonpatient questions (the factor “other factors”) were included in the actual study. The data collection procedure and study period per unit (4–6 weeks) are described earlier in the paper. Question twelve allowed nurses the opportunity to freely formulate a response to whether there are additional factors other than those listed elsewhere in the PAONCIL instrument that affect total NWL, and 2 394 comments from 75 units were seen. Of these, 1351 comments were analyzed from which the study's categories and subcategories were derived, revealing that the list of nonpatient factors included in the PAONCIL instrument was incomplete.

### 4.3. Data Analysis

Qualitative manifest content analyses were used for data analysis [26]. First, all 2 394 comments related to the factor “other factors” (additional nonpatient factors that affect total NWL) were read carefully. In that, the study focused on which nonpatient factors affect NWL; those comments clearly referring to patient-related nursing care, for example, referencing a patient's condition or a patient-related nursing situation, were excluded (1 307 comments in total). The remaining comments (*n* = 1 351) were interpreted as meaning units, with these meaning units representing expressions of additional nonpatient factors. In the first phase, the second researcher read all of the meaning units several times to get an overall view of the main content. In the second phase, both researchers condensed and coded the meaning units in relation to similarities and differences. Thereafter, the coded meaning units (codes) were categorized into subcategories. Lastly, the subcategories were finally abstracted into four categories. During the final phase, all codes were quantitatively calculated and presented for each category and subcategory.

## 5. Results

The qualitative content analyses resulted in four main categories encompassing 17 subcategories: organization of work; working conditions; self-control; cooperation (see Table 1).

### 5.1. Organization of Work

The category “organization of work” (458 comments) is composed of six subcategories: (1) the substitute nurse situation; (2) nurse manager's organization of work; (3) introduction of new staff; (4) planning of work schedules; (5) meetings and training events; (6) nursing students.

The substitute nurse situation was the most common nonpatient factor of the entire data material (211 comments) and can be described by three codes: planned number of substitute nurses; insufficient staffing resources; substitutes. The code most often mentioned as increasing total NWL was identified as insufficient staffing resources. Comments included “*once again, minimum staff*” and “*the group has only one nurse and several patients to move from surgery as well as one emergency patient!*” The second most mentioned code was substitutes. If there are sudden or unplanned absences, for example, that a nurse falls ill during a shift, it is almost impossible to find a competent substitute. Conversely, sudden or unplanned absences by physicians, requiring physician substitutes (locum tenentes), usually decreased total NWL.

Nurse manager's organization of work (*n* = 121) can be described by six codes: division of work tasks; planning; amount of work per nurse/per group; general organization of work; flow of information; administrative tasks. Participants clearly expressed a need for the purposeful organization of nursing work. A nurse manager must carefully plan each shift's skill-mix and continuously ensure that the correct competence is in place. One participant commented in regard to the planning and the general organization of work, “*I was the only nurse who could give intravenous injections during the night shift, so I had to take care of all the patients' medications.*” Participants complained that they had to care for too many patients per nurse or that they were the sole Registered Nurse (RN) working:* “to be alone and responsible for the cytostatics for ten patients.”* A lot of nursing time is taken up with the transportation of patients between units in a hospital. The participants experienced that inadequate information (“flow of information”) increased total NWL. Planned closures at outpatient clinics during holidays and so forth can decrease NWL.

Introduction of new staff (*n* = 47) was a nonpatient factor subcategory that primarily increased total NWL but could, in some cases, decrease total NWL. Some nurses found it demanding to introduce a new member of staff in addition to their usual tasks.

Planning of work schedules (*n* = 29) can be described with three codes: shorter shifts/working days; longer shifts/working days; overtime. Both shorter and longer shifts/working days seemed to stress the nursing work situation. If a working day ends, for example, after only 4-5 hours, a nurse must hurry with tasks. In some hospitals, a system of extended shifts of up to 12 hours or shifts that covered both day and night existed, which was considered stressful.

Meetings and training events (*n* = 25) factor was mentioned mostly in conjunction with outpatient clinics and was considered a positive nonpatient factor that decreased total NWL.

Nursing students (*n* = 25) can either decrease or increase total NWL: “*the supervision of the nursing students was a burden on top of the other stress, even though the student was also helping me.*” The evaluation of a nursing student's progress is often conducted on the units, which may cause extra stress for the supervising nurses.

### 5.2. Working Conditions

The category “working conditions” (396 comments) is composed of four subcategories: (1) working environment; (2) telephone traffic; (3) information technology; (4) interruptions.

Working environment (*n* = 148) included four codes: the workplace environment; equipment, and material; rooms/spaces; maintenance. The participants expressed clear disappointment with the ventilation systems at the hospitals: “*very hot on the unit, especially in the nurses' office we may have +28 degrees Celsius.*” Malfunctioning equipment or a lack of instruments caused frustration and clearly increased NWL, especially in acute patient situations: “*often problems with the alarm systems.*” Participants especially from larger organizations negatively commented on the workplace environment and maintenance, mentioning, for example, that continuous attention must be paid to stocking necessary material on each unit:* “filling cupboards.”* A lack of means, material, or other resources can cause unnecessarily stressful working conditions and increase NWL: “*I had to search for the right medication from other units*” and “*too few isolation rooms on my unit.*”

Telephone traffic (*n* = 148) in many ways increased total NWL. It was primarily participants from outpatient clinics who commented, referring to patients and patients' relatives directly contacting nurses with health problems, questions, and so forth: “*contact [information from] patients, re-scheduling appointments.*” The participants indicated that they also call patients directly in regard to care and/or treatment, care plan changes, tests, medications, and so forth.

Information technology (*n* = 68) included four codes: hardware; software (for nursing documentation or ordering laboratory tests, etc.); how IT systems worked; other IT-related equipment. Problems connected to IT appear to significantly increase total NWL.

Interruptions (*n* = 32) included three codes: self-caused interruptions (own acute illness); unplanned interruptions (changes in the organization of work); interruptions due to a colleague's absence. A work plan may include the division of nurses' time between two locations, for example,* “half the day at the out-patient clinic and the other half on the unit.”* Work colleagues can be absent because of supervision or professional development discussions and such absences clearly increase NWL. Still, interruptions can also have a decreasing effect on total NWL:* “time to breathe.”*

### 5.3. Self-Control

The category “self-control” (396 comments) is composed of four subcategories: (1) control of own work; (2) hurry/rush; (3) mental stress; and (4) own work capacity.

Control of own work (*n* = 266) can be described by three codes: primary nurse function; own competence; organization of own work. Participants described the importance of own competence in relation to organization of own work:* “I may be unsure of my own competence.”* Being the only RN in charge of an entire unit was considered a burden and the cause of mental stress: “*The (practical) nurse was not able to contribute to the care with her own share in the way she had hoped to.*” Nurses who are unable to organize their own work may feel that they do not have control over their own work, for example, if “*a part of the job [must be left] for the next shift.*”

Hurry/rush (*n* = 66) included two codes: experiencing a busy schedule; experiencing an unmanageable workload:* “no time for lunch breaks.”* The nurses described a clear link between being in a hurry and an unmanageable workload.

Mental stress (*n* = 39) included three codes: challenging patient situations; demanding nursing interventions; patient encounters, for example, in palliative care. Nurses encounter many different situations while at work, from acute resuscitation to aggressive, threatening patients:* “an aggressive patient and death threats.*” Waiting for physicians or test results in addition to uncompleted nursing interventions/tasks can cause mental stress: “*The backlog of work at the workstation ‘burdened' the mindset.*” Difficulties in accessing various test or examination results also increase the experience of mental stress.

Own work capacity (*n* = 25) included a variety of situations. It may take time for nurses to return to full work capacity after a break or a holiday and the experience of burnout or sleep disturbances may cause feelings of tiredness or fatigue: “*my own health status, I have had for about two weeks respiratory problems and a slight temperature.*” Additionally, personal or relational problems, including sorrow, worry, or anxiety, seem to have a clear effect on NWL, for example,* “if my own child is ill.”* The nurses also mentioned factors that decreased NWL, such as being in a good mood due to an upcoming holiday, a good night's rest, or* “when Finland won the Ice Hockey World Championships.”*

### 5.4. Cooperation

The category “cooperation” (101 comments) is comprised of three subcategories: (1) cooperation with physicians; (2) cooperation with other staff; and (3) cooperation with own group.

Cooperation with physicians (*n* = 47) included five codes: organization of nurse-physician cooperation; following physicians' instructions or ordinations; consultations; introductions to newly examined physicians; how experienced the physicians are. This subcategory is pivotal in nurses' experiences of NWL. How physicians organize rounds on a unit seems to be important, especially the timing and length of a round:* “the physician had to stop the round and go to another unit.”* Participants representing from every unit in this study commented on the importance of how physicians organize their work. Unclear or fluctuating instructions from physicians increase NWL: “*an operation can be cancelled, and then later on we get the information that the patient will be operated on and so on….*” Sometimes, when acute consultations are needed, nurses must wait for or call a physician many times before the physician comes to the unit.

Cooperation with other staff (*n* = 30) included two codes: nurse colleagues from other units; other professionals from supporting units, such as laboratories. These individuals can increase NWL, for example, in cases of unprofessional behavior or ineffective laboratory services: “*sometimes we have to ask for and wait for the laboratory results.*” However, successful cooperation may decrease NWL: “*we received help from the [other] unit.*”

Cooperation with own group (*n* = 24) can either decrease or increase total NWL. Participants indicated that a well-functioning and fruitful cooperation between colleagues on a unit can be a great resource: “*good work partners.*” Still, cooperation may in some cases require a nurse to divide time between his/her own patients and those of another nurse, thus increasing NWL: “*I had to help my colleague with some work tasks.*”

## 6. Discussion

During our analysis of previous research, we did not find any studies that assessed or reevaluated existing NWL instruments or scales, and we found only a few articles on the topic as a whole. Yet changes in social contexts motivate an assessment of such [27, 28]. Thus, as a consequence of the rapidly changing work context for nurses, we determined that an assessment of nurses' experiences of the nonpatient factors seen in the PAONCIL instrument was necessary. The middle and large scale hospitals included in the study were randomly selected from a list, but because there were only two small scale hospitals both were included. We maintain that the results seen here can be assessed as generalizable to, at a minimum, hospitals in Finland and even to a Nordic context, where health care is similarly organized. We understand that the size of an organization might affect nurses' experiences of those factors that affect their NWL. Larger hospitals have more specialized units, whereas smaller hospitals often have units composed of mixed patient material, which require certain flexibility with regard to nursing. The data material seen in this study, taken from 11 hospitals and 75 units, is sufficiently comprehensive and constitutes a strength of this study. Nevertheless, a deeper interpretation of the meaningful content could not be conducted because the participants' comments were in general rather short. The quantification process of the codes and each subcategory was done carefully, and we interpret these data as supplementing the qualitative results. However, the quantitative values of each category and subcategory should be considered with caution and should be understood as indicative and not absolute. While we assess that the actual results of the nurses' experiences of nonpatient factors can be generalized to an international level, this assumption should be tested further in international multicenter studies.

The results of the actual study support to a substantial extent the actual list of nonpatient factors included in the PAONCIL instrument that affect total NWL and which are seen as categories in Table 1: predominantly the first and fourth categories (organization of work and cooperation) and in part the third category (self-control). Out of a total of 1 351 comments, 458 are associated with the first category and 101 with the second category. In a recent PAONCIL study [15] the subcategory “the substitute nurse situation,” seen in the first category, was assessed as being one of the most important nonpatient factors related to NWL. The importance of well-organized and carefully planned working processes has been determined in earlier research and should be included in any future assessment of NWL [29].

On the basis of the results, the second category (working conditions) and its associated subcategories had a significant impact on total NWL. Nursing is a highly technological profession that greatly depends on various equipment, devices, and IT systems. Telephone calls clearly increase nurses' NWL in many ways. The IT systems described by the participants in the actual study were not considered to be user-friendly. The effective adoption of technology in nursing is dependent on technical skills, social acceptance, and workplace culture [30]. Continuous self-caused interruptions and interruptions due to a nurse manager's organization of work affect total NWL, but surprisingly nurses continuously dividing time between many patients or patients' relatives did not affect NWL (cf. [31]). Conflicting goals due to ongoing work tasks may have a divisive effect that increases total NWL and also consequently causes mental stress [32].

The participants frequently mentioned the subcategories included in the third category (self-control). They expressed a high level of self-awareness regarding their own competence and that they must be allowed to organize their own work. If unable to do so, nurses may not feel in control of their own work or work situation, which increases NWL. The ability to control mental stress is crucial to experiencing a work situation as being manageable, and a nurse's high competence level may ease a stressful situation. According to Schmidt [33], improving the “fit” between personal and organizational goals and strengthening an individual's control resource could make health care workers less vulnerable to the depleting effects of meeting self-control demands at work. Furthermore, a work situation that does not give nurses time for lunch breaks can be considered a clear health threat. There is an evident connection between how nurses feel and their performance and nursing outcomes [34]. Nursing in general is described as stressful work that often leads to a constant hurry/rush with mental stress as a consequence. Most of the comments about mental stress seen in the data were related to demanding encounters and actions with patients or patients' relatives. Nurses are also experiencing increasing violence [35]. Due to threatening situations, many organizations have hired security staff. Even working nights, evenings, or weekends cause mental stress in nursing [36]. Also, personal lifestyle and conditions at home may have a negative effect on nurses' work capacity, clearly seen in the actual data. The importance of well-organized and well-planned work processes and the need for a work-life balance have been found in earlier research and as such should be taken into consideration [28].

The fourth category (cooperation) included three subcategories. All forms of cooperation are highly valued by nurses (cf. [15, 24]). In the actual study, participants commented on cooperation with physicians, including organization of the nurse-physician cooperation, following physicians' instructions or ordinations, consultations, introductions to newly examined physicians, and how experienced the physicians are. Cooperation entails a synergistic alliance that maximizes each member's contribution to an entity [37]. Effective cooperation requires a connection to a common goal, collegiality, and good communication [38]. Hierarchal levels that favor the medical profession have a negative effect on the results of health care (cf. [39]).

There is an increasing recognition that both patients and staff exist in an organizational environment and that the features of that environment have a clear impact on nurses' job satisfaction [13, 29, 40]. Nurses' job satisfaction is therefore closely related to working conditions. Factors that affect working conditions include the organizational environment, job stress, role conflict and ambiguity, role perception and role content, and organizational and professional commitment [40]. In addition, continuous stress and “the work-home interface” have been found to have a clear influence on turnover for nurses [40, 41]. A recent Irish study showed that stress due to an excessive workload continues to be a problem for nurses [42]. Suggested interventions for improving the clinical environment should focus on NWL, working relationships, and clinical learning needs. Negative working conditions, job stress, and job dissatisfaction lead to higher levels of absenteeism among nurses, and work environment factors that increase NWL should be carefully considered by nurse managers [43]. Still, good cooperation between colleagues may decrease total NWL. Social support given by work colleagues may lower nurse students' and nurses' job stress and increase job satisfaction [44]. A more favorable nurse work environment is associated with better nurse outcomes, such as lower levels of burnout, overall stress, job dissatisfaction, and intention to leave [45, 46]. Providing a good professional practice environment for staff nurses has been one central goal of the Magnet programs [47].

Surprisingly, few comments in the actual study described nonpatient factors that could decrease total NWL. A good quality nursing work environment is influenced by nursing resources (quantity and quality), a supportive leadership style, the level of nurses' autonomy and decision making, cooperation and teamwork, and organizational culture [48]. Earlier research has shown that an NWL that exceeds the optimal level leads to lowered individual work capacity [49, 50]. Therefore, an optimal NWL, which allows the individual nurse to feel that his/her work situation is manageable and that working conditions support good nursing care and patient outcomes, is crucial to preventing sick leaves [51], for job satisfaction and for the recruitment of staff.

The health care sector's economic situation is deteriorating, and nurse managers face a great challenge in balancing nursing resources and patients' needs and ensuring optimal working conditions. By using the RAFAELA system to systematically measure NWL (NI and nonpatient factors), nurse managers can obtain evidence of which factors threaten an optimal work environment [52]. Central factors for nurses' satisfaction with their psychosocial work environment are work stress, cooperation, good collegial communication, job motivation, work demands that incorporate ethical demands, and professional development [52].

Future research should focus more on discovering which factors decrease NWL. Given that many organizations today are larger than ever, what can be done to support good interprofessional and interunit cooperation and what can be done to prepare future nurses for such a demanding work environment should be investigated. The nursing work environment seems to be more demanding than ever, and the hiring of more staff cannot always solve the problems associated with high NWL (cf. [53, 54]). It is therefore critical to discover how nurses' ability to manage work stress can be developed and supported. Nurse managers are responsible for the overall work situation on a unit, and more research is needed on how nurse managers can strengthen the nursing work environment and support nurses. Additionally, more knowledge of what each nurse can do to improve his/her own self-control, commitment to work, professional development, work health, and well-being is also needed (cf. [52]).

## 7. Conclusions

While the actual list of nonpatient factors in the PAONCIL instrument is to a reasonable extent relevant, the list should nonetheless still be improved. The second and third categories (working conditions and self-control) appeared to highlight new dimensions of nonpatient factors that have not been sufficiently explored in nursing research. Our results showed a need to capture other factors related to a modern view of the second category (working conditions) and all of the different dimensions of the third category (self-control) and showed that the actual list of nonpatient factors in the PAONCIL instrument should be renewed. Nurse managers must focus on and develop own competence in the steering of NWL, including control and follow-up of both NI and nonpatient factors, if unnecessary absences and sick leaves are to be avoided. This will also help increase staff retention and job satisfaction and improve patient outcomes. Evidence-based tools, methods, and PCS such as RAFAELA are clearly needed. However, making the work environment manageable is not only a task for nurse managers; nurses must also develop their self-control. Each staff member must contribute to effective cooperation on all levels and develop an awareness of how a good work-life balance can be achieved.

## Figures and Tables

**Table 1:** Nonpatient factors that affect total NWL, described as categories and subcategories.

Organization of work 458 comments	Working conditions 396 comments	Self-control 396 comments	Cooperation 101 comments
The substitute nurse situation (211 c∗)	Working environment (148 c)	Control of own work (266 c)	Cooperation with physicians (47 c)

Nurse manager's organization of work (12 c)	Telephone traffic (148 c)	Hurry/rush (66 c)	Cooperation with other staff (30 c)

Introduction of new staff (47 c)	Information technology (68 c)	Mental stress (39 c)	Cooperation with own group (24 c)

Planning of work schedules (29 c)	Interruptions (32 c)	Own work capacity (25 c)	

Meetings and training events (25 c)			

Nursing students (25 c)			

*c: comments.
